# Data-Driven Early Warning Approach for Antimicrobial Resistance Prediction–Anomaly Detection Based on High-Level Indicators

**DOI:** 10.3390/vetsci12100935

**Published:** 2025-09-26

**Authors:** Szilveszter Csorba, Krisztián Vribék, Máté Farkas, Miklós Süth, Orsolya Strang, Andrea Zentai, Zsuzsa Farkas

**Affiliations:** 1 Department of Digital Food Science, Institute of Food Chain Science, University of Veterinary Medicine, H-1078 Budapest, Hungary; csorba.szilveszter@univet.hu (S.C.); vribek.krisztian@univet.hu (K.V.); farkas.mate@univet.hu (M.F.); strang.orsolya@univet.hu (O.S.); farkas.zsuzsa@univet.hu (Z.F.); 2 National Laboratory of Infectious Animal Diseases, Antimicrobial Resistance, Veterinary Public Health and Food Chain Safety, University of Veterinary Medicine Budapest, István utca 2., H-1078 Budapest, Hungary; suth.miklos@univet.hu; 3 Institute of Food Chain Science, University of Veterinary Medicine, H-1078 Budapest, Hungary

**Keywords:** AMR, environmental drivers, one health, iForest, SHAP

## Abstract

**Simple Summary:**

Antimicrobial resistance (AMR) is a growing threat to human and animal health, and while environmental conditions might play a role in its spread, it is not yet explored. However, detecting risky environmental conditions early is difficult. In this study, we used a computer-based method to spot unusual patterns in environmental data, such as pesticide use, rainfall, and farming practices. We also analyzed which factors contributed most to the unusual patterns that might lead to increased AMR risk. Our results showed that pesticide use, changes in land use, population density, and fertilizer application were the most important drivers from the examined environmental conditions. Extreme weather and production of certain crops also played a role in some cases. This new approach helps in creating early-warning monitoring systems, mitigation and preparedness and through that, protecting public health.

**Abstract:**

Environmental conditions are increasingly recognized as important contributors to the emergence and spread of antimicrobial resistance (AMR), yet early detection of high-risk situations remains difficult. This study developed a data-driven framework to identify anomalous environmental profiles associated with potential AMR risk. Using an unsupervised anomaly detection method (Isolation Forest) applied to multivariate indicators—including pesticide use, land use change, precipitation, and crop type—we detected unusual environmental patterns without prior AMR data. The anomaly detection analysis highlighted pesticide use, population density, land use change, and fertilizer application as the dominant environmental factors, together explaining the largest share of variation in anomaly scores (each contributing around one-quarter to one-third of the model’s decisions). In the subset of anomalous cases, fertilizer and pesticide intensity exerted the strongest negative impact, confirming their role as key drivers of atypical environmental profiles. Extreme precipitation and crop-specific production patterns also emerged as influential in certain cases. These results show that our interpretable framework can both rank global drivers and reveal context-dependent risks, thereby enabling the development of early-warning strategies for AMR surveillance.

## 1. Introduction

Antimicrobial resistance (AMR) represents one of the most pressing global health challenges, undermining the efficacy of antibiotics and treatments for infectious diseases. The rise in resistant pathogens leads to prolonged illnesses, higher mortality, and escalating healthcare costs worldwide [1]. AMR arises through genetic mutations and horizontal gene transfer, enabling bacteria to evade antimicrobials. Its development is driven mainly by antibiotic misuse in human medicine (e.g., inappropriate prescribing for viral infections) and agriculture (e.g., growth promotion or prophylaxis in livestock), which facilitates resistance spread via direct contact, food, and environmental pathways [2,3]. As a One Health issue compounded by globalization, AMR demands coordinated action across human, animal, and environmental sectors [4].

While the overuse and misuse of antibiotics in human medicine, veterinary practices, and livestock production are widely recognized as primary drivers of AMR, the environmental dimension of this crisis is increasingly being acknowledged as a critical factor in the dissemination and persistence of antibiotic resistance genes (ARGs) [5]. The environment acts as a vast reservoir and transmission pathway for ARGs, with agricultural practices, climate change, and land use changes playing pivotal roles in shaping the dynamics of AMR [6,7].

Agricultural activities, particularly the application of fertilizers, are among the contributors to the spread of AMR in the environment. Nitrogen-based fertilizers, which are widely used to enhance crop yields, have been shown to influence the abundance and diversity of ARGs in soil ecosystems by creating selective pressures that favor the survival and proliferation of resistant bacteria [8]. The cultivation of staple crops, which are integral to global food security, further exacerbates this issue by intensifying land use and altering soil microbiomes [9]. The production of crops, which are often grown in monoculture systems, can lead to soil degradation and reduced biodiversity, further exacerbating the environmental pressures that drive AMR [10,11,12]. Additionally, the use of pesticides and herbicides in crop production has been shown to co-select for antibiotic resistance [13,14]. These changes, combined with the widespread use of antibiotics in livestock and crop production, create a complex interplay between agricultural practices and environmental AMR. As global demand for food continues to rise, the intensification of agricultural systems is likely to amplify these pressures, making it imperative to explore sustainable practices that minimize the environmental impact of AMR [12].

Climate change, characterized by rising global temperatures and altered precipitation patterns, is another critical factor influencing the environmental spread of AMR. Higher temperatures have been linked to increased bacterial growth rates and enhanced horizontal gene transfer, accelerating the proliferation of ARGs [15,16]. Furthermore, extreme weather events, such as heavy rainfall and flooding, can facilitate the transport of antibiotic-resistant bacteria and ARGs from agricultural fields into water bodies, contaminating renewable water resources [17]. This is particularly concerning given the reliance on these resources for drinking water, irrigation, and recreational activities [18]. The contamination of water resources with ARGs poses a direct threat to human health, as it increases the likelihood of exposure to resistant pathogens through various pathways.

The interplay between land use changes, crop production, and greenhouse gas (GHG) emissions further complicates the environmental impact of AMR. Intensive agricultural practices not only contribute to GHG emissions but also alter soil and water ecosystems, creating environments conducive to the persistence and spread of ARGs [19]. For instance, the conversion of natural habitats into agricultural land can affect soil health and characteristics changing microbial communities and increases the likelihood of ARG transfer between environmental and pathogenic bacteria [11,20]. As the global population continues to grow, the demand for food and agricultural products is expected to intensify, highlighting the need for sustainable land use practices that balance productivity with environmental preservation [21]. Understanding the environmental drivers of AMR is essential for developing comprehensive strategies to mitigate its spread and safeguard public health.

Addressing the environmental drivers of AMR requires a multidisciplinary approach that integrates insights from microbiology, environmental science, agriculture, and public health, ensuring that efforts to combat AMR are both effective and sustainable.

Given these complex interactions, there is a pressing need for a systematic approach to analyze the environmental factors influencing AMR. Utilizing data analytical methods can provide valuable insights into the relationships among various environmental variables.

Anomaly detection methods—such as Isolation Forest (iForest)—are designed to flag data points that significantly deviate from expected patterns [22]. These multivariate approaches are widely used across various domains including fraud detection to identify abnormal financial transactions based on multiple features [23]; cybersecurity to detect network intrusions [24], and environmental sensor networks. In the latter scientific field, multivariate anomaly detection frameworks have been demonstrated to effectively identify unusual combinations of environmental variables (e.g., changes in mean, variance, seasonal trends) compared to univariate methods [25]. Moreover, in agricultural and land--use monitoring, researchers have applied iForest to satellite imagery to detect anomalies such as wildfire burn zones or quarry expansion with accuracies of 95–98% [26]. By developing a multivariable anomaly detection model that incorporates fertilizer use, precipitation, temperature, land use, and crop yield, future analyses can more precisely detect environmental conditions that significantly influence AMR dynamics.

This study aims to establish a framework for environmental multivariable anomaly detection related to AMR, laying the groundwork for future research that can quantitatively assess how these environmental factors contribute to the emergence and spread of antibiotic resistance. Understanding these dynamics is essential for creating effective strategies to mitigate the environmental impact of AMR and ultimately protect public health.

## 2. Materials and Methods

### 2.1. Overall Early Warning Framework

**Figure 1:**
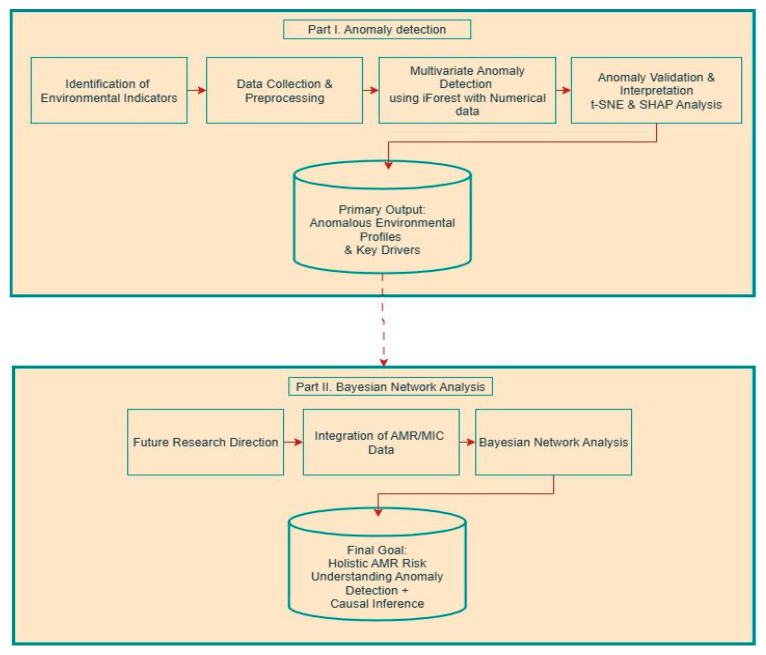
Schematic of the two-stage analytical pipeline. Part I (this study) details the unsupervised anomaly detection process applied to environmental data. Part II (ongoing work) outlines the planned integration of AMR data and Bayesian analysis to quantify causal relationships and achieve a holistic risk model.

A schematic overview of the complete early warning framework is presented in Figure 1. This research is conceived as a two-part framework designed to progressively address the environmental drivers of Antimicrobial Resistance (AMR). The current study (Part I) constitutes a foundational, unsupervised analysis focused solely on identifying anomalous patterns within a multivariate set of environmental indicators. The subsequent phase (Part II), ongoing research soon to be published, builds upon these findings by integrating AMR data to construct a predictive Bayesian model.

The workflow begins with the identification of environmental indicators (detailed in Section 2.2) based on their potential to influence AMR dynamics. These indicators are then subjected to a rigorous process of data collection and preprocessing (Section 2.3) from global databases to create a unified, clean numerical dataset.

The core of Part I is multivariate anomaly detection using the iForest algorithm (Section 2.5). This unsupervised method is applied to numerical environmental data to identify observations that significantly deviate from the expected multivariate pattern without the use of AMR data.

These results provide the critical foundation for Part II. The planned future research will take the key drivers identified here and perform an integration with AMR/MIC (Minimum Inhibitory Concentration) data, using it as a target variable. A Bayesian Network Analysis will then be employed to quantify the probabilistic relationships and contributions of the environmental drivers to AMR prevalence and behavior.

The final goal of this overarching framework, presented in Figure 1. is to achieve a holistic understanding of AMR risk by combining the anomaly detection capabilities of Part I with the causal inference power of Part II, ultimately informing the development of a data-driven early-warning system.

### 2.2. Identification of Environmental Indicators

The identification of environmental indicators was a first step in the exploration of the complex interactions between climate change, crop production, anthropogenic activities, pests, diseases, and AMR. These indicators were selected based on their potential to significantly impact the AMR, either directly or indirectly. While direct metrics of broiler production are crucial, this study focused on broader environmental and anthropogenic indicators that act as precursors to AMR risk. Variables such as maize and rapeseed yield were selected as high-level proxies for intensive agricultural activity due to their global data availability and strong association with agrochemical input. The process involved a multidisciplinary approach, combining insights from experts in environmental science, agriculture, microbiology, and epidemiology, as well as an extensive review of existing scientific literature and data sources.

### 2.3. Data Collection and Sources

The indicators we considered relevant were collected from reputable sources which are authoritative databases providing global statistics on agriculture, food, and the environment (Table 1). This collection of datasets included a variety of variables relevant to the study, such as agricultural practices, land use, water resources, and other environmental metrics that could potentially influence AMR trends. While the source databases provided historical data (e.g., from 1961), for this analysis we extracted a consistent modern time series from 1990 to 2022 for all variables to ensure temporal coherence and relevance to contemporary agricultural practices.

Data for the selected indicators were obtained from international database collections (FAOSTAT, EuroSTAT, Our World in Data). Whenever possible, we prioritized high-resolution, long-term datasets to capture both spatial and temporal variability. The integration of multiple data sources allowed for a more comprehensive analysis of the interactions between indicators.

For regional analyses, we incorporated site-specific data provided by local agricultural agencies and research institutions. This facilitated the identification of localized patterns and trends, which are often masked in global datasets. In cases where direct measurements were unavailable, proxy variables such as land use which influences natural habitat loss, and biodiversity that potentially affect AMR indirectly were used to estimate the values of certain indicators.

### 2.4. Feature Engineering and Data Preparation

To create a unified dataset, we performed data merging using systematic data wrangling techniques. This process involved aligning the used environmental data, specifically country codes and time periods (e.g., year). By using these common keys, we ensured that the datasets were correctly matched and no critical information was lost during the integration process. This step was pivotal in combining the 10 indicators’ datasets (Table 1) into a single, structured data table that could be used for downstream analysis.

Following the data merging process, we addressed the issue of missing values, which is a common challenge when working with large, heterogeneous datasets. Missing data can arise for various reasons, such as incomplete reporting, data collection errors, or differences in coverage across databases. To handle this, we employed Iterative Imputation (also known as Multivariate Imputation by Chained Equations or MICE) data imputation techniques to minimize the impact of missing values on the quality and reliability of the dataset. MICE is a robust, flexible technique for imputing missing data by modeling each variable with missing values as a function of other variables, iteratively refining the imputations. It is widely supported in statistical software like R (mice package, version 3.16.0) [27] and Python (version 3.16.0). The entire data wrangling, merging, and imputation process was conducted in Python using pandas for data manipulation and the sklearn.impute. IterativeImputer class from the scikit-learn library (v1.2+) for missing data imputation [28].

### 2.5. Anomaly Detection Using iForest

For the unsupervised identification of anomalous environmental profiles, we employed the iForest algorithm [28].

**Figure 2:**
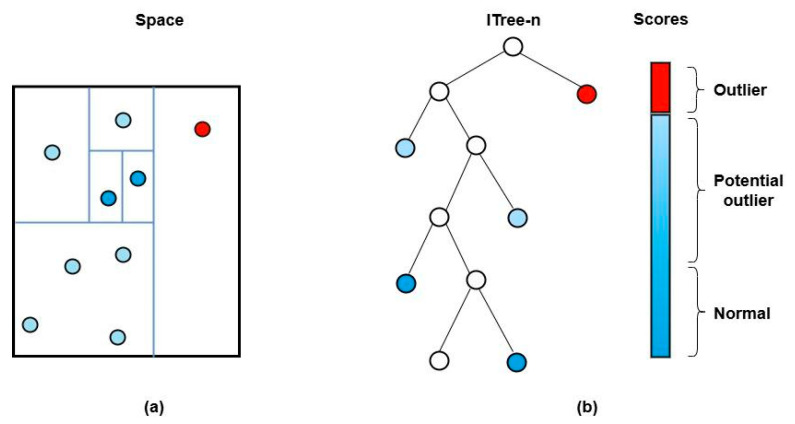
iForest data processing procedure. (**a**) Data segmentation method: The algorithm recursively partitions the data by randomly selecting a feature and a split value between its current minimum and maximum. (**b**) Isolation tree construction: This process builds an isolation tree. Outliers (represented by the red circle) are rare and distinct data points that are isolated with fewer splits, resulting in a shorter path length. Normal data points (represented by the dark blue circle) are more densely distributed and require more splits to be isolated, resulting in a longer path length. The algorithm flags data points with very short average path lengths across many trees as potential outliers (represented by the light blue circle) [29].

The core principle of iForest is that anomalies are few, distinct, and therefore easier to isolate from the majority of data (Figure 2). The algorithm builds an ensemble of binary trees (isolation trees, or iTrees) using a random partitioning process. In each tree, data are recursively partitioned by randomly selecting a feature and then a random split value between the observed minimum and maximum of that feature. The number of splits required to isolate a single data point—its path length—is inversely proportional to its anomaly degree; anomalous points are isolated with significantly fewer splits than normal points. 

The model was configured with the following hyperparameters:

- n_estimators = 100: Number of isolation trees in the ensemble.
- max_samples = “auto”: The number of samples used to build each tree (default: 256).
- contamination = 0.03: The proportion of outliers in the dataset (3%).
- random_state = 42: Seed for reproducibility.

The decision threshold for classifying a data point as anomalous was automatically determined based on the specified contamination rate. This threshold defines the cutoff point for the anomaly score, distinguishing between normal and anomalous instances. Internally, this corresponds to a density threshold, where data points in low-density regions (as estimated by techniques such as Kernel Density Estimation, KDE) are flagged as anomalies, while those in high-density areas are considered normal.

This unsupervised method leverages the iForest algorithm’s intrinsic capacity to detect deviations from general data patterns without requiring labeled input. As such, it is particularly effective for exploratory analysis, enabling the identification of rare or atypical observations within a dataset.

### 2.6. t-SNE Analysis for Anomaly Score Validation

To validate the anomaly scores generated by the iForest model, t-distributed Stochastic Neighbor Embedding (t-SNE) was employed [30]. This dimensionality reduction technique allows for the visualization of high-dimensional data in a lower-dimensional space (typically two or three dimensions), making it easier to identify clusters and anomalies. The steps for t-SNE analysis included:

Input preparation: The anomaly scores along with the original feature set were prepared as input for the t-SNE algorithm.

Visualization: The resulting t-SNE embeddings were plotted to visualize the distribution of normal and anomalous instances, facilitating a qualitative assessment of the model’s performance in detecting anomalies.

### 2.7. SHAP-Based Model Interpretation

While the Isolation Forest algorithm effectively identifies anomalous observations, it lacks inherent mechanisms to explain why a specific point was flagged as an anomaly. To overcome this “black box” limitation and extract meaningful, actionable insights from our model, we employed SHAP (SHapley Additive exPlanations) values. SHAP is a unified framework based on cooperative game theory that assigns each feature an importance value for a particular prediction. Its core advantage is that it satisfies properties desirable for explanations (local accuracy, consistency, and missingness), providing a theoretically sound and consistent measure of feature contribution. SHAP provides both global (model-wide) and local (instance-level) interpretability by approximating the marginal contribution of each feature to the final prediction.

Global Interpretability (Model-Level): To understand the overall drivers of anomalies across the entire dataset, we calculated the mean absolute SHAP value for each feature. This provides a robust ranking of which environmental indicators, on average, have the largest impact on the model’s anomaly detection decisions.

Local Interpretability (Population-Level): To explore the directionality and distribution of each feature’s effect, we generated a beeswarm plot. This plot visualizes the SHAP value for every feature and every observation in the dataset. The position on the *x*-axis shows the impact (negative or positive) on the anomaly score, and the color represents the actual value of the feature (from low in blue to high in red). This allows for the identification of non-linear relationships and general trends (e.g., “high pesticide use consistently increases the anomaly score”).

Local Interpretability (Instance-Level): To deconstruct the precise reasoning behind the classification of specific anomalous instances, we generated waterfall plots. These plots start from the baseline expected value and sequentially add each feature’s SHAP value, showing how the combination of feature values led to the final anomaly score for a single observation or a group of similar anomalies. This is critical for domain experts to validate and understand individual high-risk cases.

All SHAP computations were performed on the standardized feature set used to train the iForest model to ensure consistency. This interpretability framework transforms the unsupervised anomaly detection results into a diagnosable tool for identifying the key environmental factors associated with high AMR risk. The SHAP computations were performed using the shap library in Python [31,32], adapted to tree-based ensemble methods.

## 3. Results

### 3.1. Anomaly Detection via iForest

**Figure 3:**
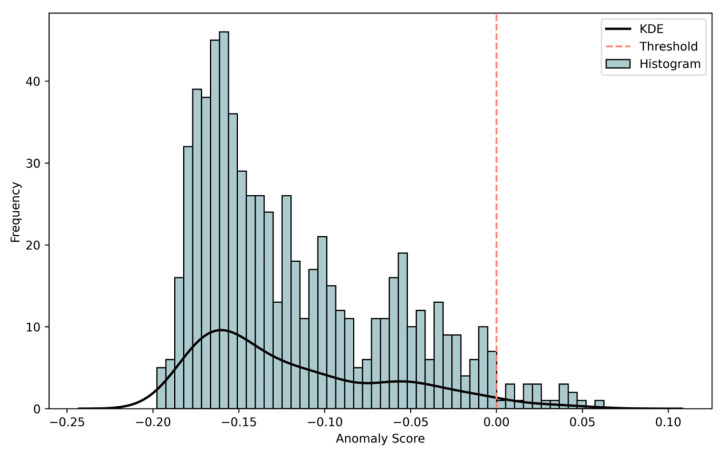
Distribution of anomaly scores generated by the Isolation Forest model. The histogram shows the frequency of data points across different anomaly scores, with a kernel density estimate (KDE) curve overlaid. The majority of observations cluster around scores between −0.15 and 0.00, representing normal environmental profiles. The distribution is right-skewed, with a long tail of potential anomalies (scores > 0). The vertical red dashed line indicates the decision threshold (≈ −1.756 × 10^−17^) for classifying anomalies, corresponding to a contamination rate of 3.06%.

The iForest algorithm was applied to identify atypical environmental patterns potentially associated with antimicrobial resistance (AMR) emergence. In the initial phase, we analyzed the distribution of anomaly scores produced by the algorithm. As shown in Figure 3, most observations cluster around scores between −0.15 and 0.00, representing normal data points that require typical path lengths for isolation. The distribution reveals a right-skewed pattern, with the majority of data concentrated around moderate anomaly levels and a smaller tail of extreme anomalies. The long right tail extending toward positive values identifies potential anomalies that were unusually easy to isolate.

With a contamination level of 0.03 the model identified 21 anomalous observations out of the total dataset. The optimal threshold for anomaly detection was determined as −1.756 × 10^−17^, indicated by a vertical red dashed line in Figure 3. This threshold effectively separates the main distribution from outlier points, capturing approximately 3.06% of the data as potential anomalies while maintaining a reasonable false positive rate. The few observations with anomaly scores exceeding 0.05 represent particularly strong anomaly candidates warranting further investigation.

The KDE curve reinforces the right-skewed pattern, emphasizing the separation between typical observations (left side) and rare anomalies (right side).

### 3.2. Validation with t-SNE

The t-SNE projection into three dimensions confirmed that anomalous observations were clustered separately in the embedded space, reinforcing the iForest’s ability to capture meaningful outliers.

**Figure 4:**
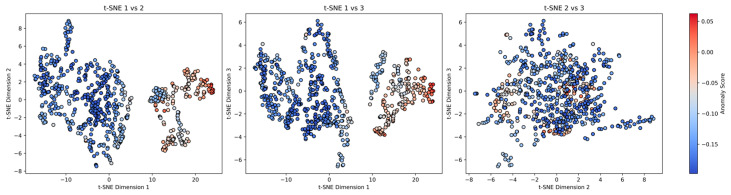
Validation of anomaly detection results using pairwise 2D projections from a 3D t-SNE embedding. Each scatter plot represents two dimensions of the reduced 3D space. Data points are colored by their iForest anomaly score (see gradient bar at the right). The dense, central cluster of blue/green points represents normal observations with low anomaly scores. The isolated red and orange points, scattered in the peripheral and low-density regions of the plots, are the anomalies identified by iForest. Their spatial separation from the main cluster confirms they are meaningful outliers and not artifacts of the data structure.

To evaluate anomalous patterns in the multidimensional dataset, we applied iForest for unsupervised anomaly detection, followed by a 3D t-SNE projection to visualize the distribution of anomaly scores. Pairwise 2D projections of the t-SNE space (Figure 4) revealed the spatial structure of the anomaly scores across three dimensions.

The results show that the majority of data points exhibit consistently low anomaly scores (in the range of −0.20 to the selected threshold), forming a coherent and dense manifold in the t-SNE space. These points represent the baseline behavior of the system. In contrast, a subset of data points, distributed irregularly throughout the projection, presented significantly elevated anomaly scores. These high-scoring outliers did not form contiguous clusters nor concentrate within specific index ranges, indicating that they are not artifacts of systematic data issues but are more likely genuine and context-specific anomalies.

Notably, several distinct subregions in the t-SNE space, particularly along peripheral and low-density zones, contained the most extreme anomalies. These points, characterized by their red coloring in the score gradient, represent the strongest candidates for further investigation. Their spatial isolation from high-density regions of typical observations underscores their potential relevance as non-random or event-driven deviations.

This analysis confirms the effectiveness of the iForest in identifying rare but critical deviations in the dataset. The t-SNE projection further enhances interpretability by revealing hidden structures and potential causal pathways for anomalies relevant to antimicrobial resistance, warranting future domain-specific and temporal tracing.

### 3.3. SHAP-Based Feature Interpretation

#### 3.3.1. Global Feature Interpretation

**Figure 5:**
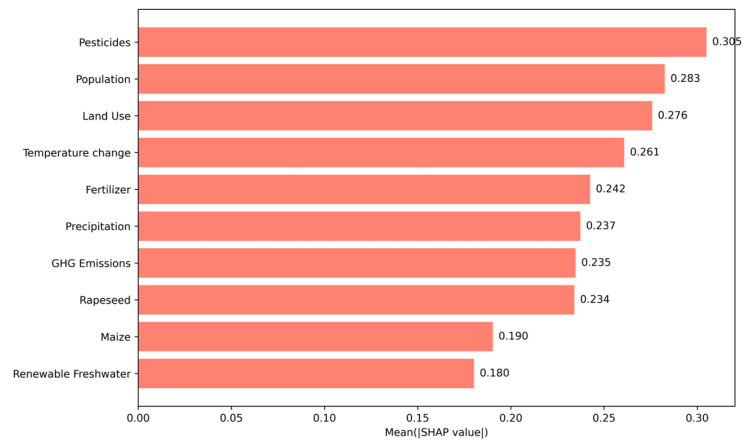
Global Feature Importance of Environmental Indicators Based on Mean ISHAP Values. The plot ranks the environmental indicators by their average contribution to the anomaly scores across the entire dataset. Pesticide Use, Population Density, Land Use, and Temperature Change were the most influential drivers in identifying anomalous environmental profiles. Features are sorted from top to bottom in descending order of importance.

We evaluated the overall impact of environmental variables on anomaly detection using mean absolute SHAP values. As shown in Figure 5, the top contributors to anomaly scores were pesticide usage, population density, land use change, and temperature change. Pesticides exhibited the highest mean SHAP value (~0.31), suggesting a strong and consistent influence on the model’s anomaly decisions. These findings highlight the central role of anthropogenic pressure—especially agrochemical input and population-driven land transformation—in shaping atypical environmental profiles.

Secondary contributors included fertilizer use, precipitation, and greenhouse gas (GHG) emissions, reflecting both climatic and agricultural influences. Variables such as rapeseed, maize, and renewable freshwater appeared with lower importance scores, indicating less consistent impact on global model behavior.

#### 3.3.2. Local SHAP Distribution and Directionality of Effect

**Figure 6:**
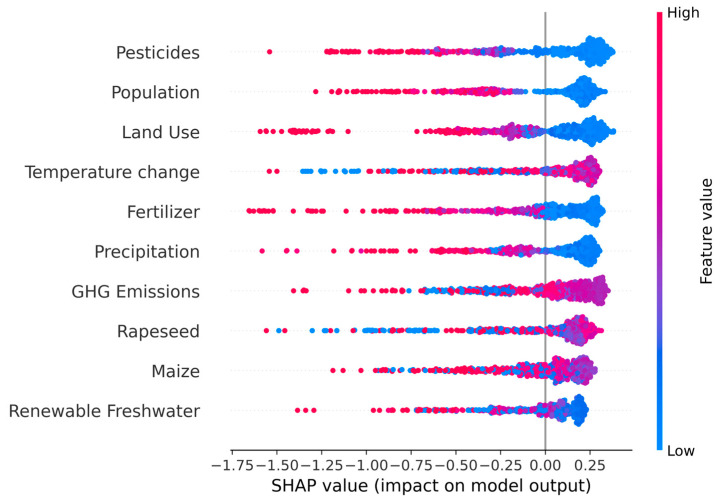
Local Feature Importance Analysis Using SHAP Values (Impact on Model Output). Each point represents a single observation from the dataset. The position on the *x*-axis is the SHAP value (negative values increase the anomaly score), and the color indicates the feature value for that observation (red = high, blue = low). For key drivers like Pesticide Use and Population Density, high values (red) are consistently associated with negative SHAP values, pushing predictions towards anomaly. The plot reveals nuanced relationships, such as high Precipitation (red) sometimes contributing to anomalies, contrary to initial expectations.

To explore how feature values influence the direction and magnitude of anomalies, we examined a SHAP beeswarm plot (Figure 6). SHAP values were interpreted based on their sign (negative values increase anomaly score; positive values decrease it) and magnitude. Pesticides showed the strongest directional effect: high pesticide values (red) were consistently associated with negative SHAP values, confirming their role in driving environmental anomalies. High population density and land use followed a similar pattern. However, land use SHAP values were clustered around the center, indicating a stable but weaker effect compared to the previous two features. Interestingly, precipitation showed a more nuanced pattern: contrary to initial expectations, low precipitation values (blue) were mostly neutral, while high precipitation (red) values sometimes pushed the anomaly score downward, suggesting that extreme rainfall events may contribute to anomaly formation. Fertilizer and temperature effects were less uniform, suggesting non-linear or context-specific influences.

#### 3.3.3. Feature-Level Interpretation Using SHAP Value Ranges

To further assess the role of each feature, we systematically analyzed their SHAP value distributions using a quantitative scale. SHAP values were classified as strongly negative (≤−1.0), moderately negative (−1.0 to −0.5), mildly negative (−0.5 to −0.2), or neutral (−0.2 to +0.2). This classification provided a consistent framework for comparing variables.

Pesticides, land use and population density showed strong to moderate negative SHAP values for high feature values, confirming them as consistent anomaly drivers.

Precipitation demonstrated only mild negative SHAP contributions, limited to specific contexts (e.g., extreme rainfall).

Temperature change, fertilizer and GHG emissions exhibited mixed effects, depending on the interaction with other variables.

Crop-related variables (e.g., maize, rapeseed, renewable freshwater) displayed low global impact but meaningful local contributions in isolated cases.

This stratified SHAP interpretation confirms that anomaly behavior is driven by a combination of dominant global factors and context-specific environmental interactions.

#### 3.3.4. Waterfall SHAP Analysis of Anomalous Samples

**Figure 7:**
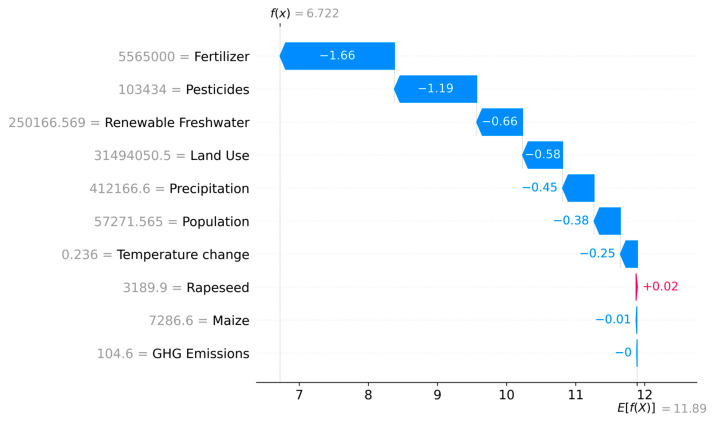
Waterfall Plot of SHAP Values for Anomalous Samples. The plot decomposes how each feature contributes to the final anomaly score for anomalous observations. The baseline value, E[f(X)], is the expected model output. Each bar shows the mean negative SHAP value (reducing the model output from the baseline) for each feature within the anomaly group. Fertilizer, Pesticides, and Renewable Freshwater were the top contributors to the anomalous classification in this subset. The final value, f(x), is the average predicted anomaly score for these samples.

To complement the global and local interpretations, we generated a SHAP waterfall plot summarizing the average feature contributions across the anomalous samples (i.e., samples with anomaly score > 0). SHAP waterfall plot for the selected anomalous samples showing the additive contribution of each feature to the final anomaly score predicted by the iForest model. Negative SHAP values (blue bars) increase the anomaly score, while positive values (red bars) reduce it. The baseline value (E[f(X)]) represents the average model output across the dataset, and the final prediction (f(x)) corresponds to the observation-specific anomaly score. (Figure 7). The reduction in the model output (f(x)) can be primarily attributed to several variables exhibiting substantial negative SHAP values. In this case, the predicted anomaly score was f(x) = 6.72, which is lower than the dataset’s baseline value E[f(X)] = 11.89, suggesting anomalous observation. The plot reveals how each feature contributed negatively to the model output, thereby increasing the sample’s anomaly severity relative to the baseline.

The most impactful features were fertilizer (−1.66), pesticides (−1.19), and renewable freshwater (−0.66), followed by land use (−0.58), precipitation (−0.45), and population (−0.38). These variables contributed negative SHAP values, thereby lowering the model’s prediction relative to the expected value—consistent with stronger anomaly classification.

Interestingly, some features (e.g., precipitation, renewable freshwater) that were only mildly impactful in the full dataset emerged as important within the anomaly subset—highlighting their context-dependent role. Other variables, such as GHG emissions, maize, and rapeseed, showed minimal or even slightly positive SHAP values on average, indicating little to no role in this subset.

This targeted analysis validates the consistency of pesticides use and fertilizer input as strong anomaly drivers and demonstrates the importance of local interpretation in uncovering hidden contributors.

## 4. Discussion

This study aimed to identify and interpret environmental anomalies associated with potential antimicrobial resistance (AMR) risk using an unsupervised machine learning approach. By applying the iForest algorithm alongside SHAP-based interpretability techniques, we were able to assess not only which environmental indicators drive anomalies, but also how their influence varies across samples.

Our findings consistently identified pesticide usage, fertilizer input, land use change, and population density as the strongest contributors to anomalies. The global SHAP summary plot revealed that these features had the highest average impact across the entire dataset, while the beeswarm plot further confirmed their directional effect—particularly showing that high pesticide levels and dense population concentrations strongly pushed the model toward anomalous classification.

The analysis also uncovered the context-specific importance of several variables. For example, precipitation did not emerge as a top global driver but had significant influence on certain anomalous samples. Contrary to expectations that low precipitation would signal environmental stress, it was high precipitation—likely reflecting extreme hydrological events such as flooding—that was more often associated with anomalies. This highlights the importance of capturing non-linear relationships that may not be evident in traditional correlation analyses.

Another important insight emerged from the SHAP waterfall plot, which decomposed the SHAP values for group of the anomalous samples. These visualizations demonstrated how the average SHAP values were reduced from the model’s expected baseline by the negative contributions of specific features. In particular, renewable freshwater, which had a moderate influence global SHAP value, emerged as a substantial local driver of anomalies in this subset—suggesting that crop-specific stressors or imbalances may be critical in specific geographic area (e.g., a drought-prone region, a watershed).

The use of SHAP values proved essential in overcoming one of the main limitations of iForest: the lack of native interpretability. By quantifying the contribution of each feature to each prediction, SHAP enabled a transparent and meaningful interpretation of anomaly scores. This capability is especially valuable for environmental monitoring and policy decision-making, where understanding why an area is classified as anomalous is just as important as identifying that it is.

Furthermore, the ability to contrast global patterns with sample-specific behaviors highlights the importance of multi-scale interpretability. Features related to specialized crop cultivation, which showed minimal global influence, nonetheless had notable effects in specific cases. This suggests that effective AMR early-warning systems must account for both widespread drivers and localized risks.

### Limitations and Future Directions

While the current framework offers valuable insight, we acknowledge its limitations, which also define clear pathways for future research. The primary limitation is that the model was trained solely on environmental indicators. Although their selection was grounded in expert knowledge and literature, the lack of direct microbiological or AMR data means the link between detected anomalies and elevated AMR prevalence remains a testable hypothesis rather than a validated conclusion.

This limitation is fundamentally addressed by the iterative design of our research pipeline. The findings from the causal analysis in Part II will provide the necessary validation and direct feedback to refine the Part I model. Specifically, through the application of machine learning feature importance techniques and Bayesian structure learning, Part II will interrogate the data’s underlying causal architecture. The outputs of this exploration—including the identification of new critical variables (e.g., global production and consumption data on boilers, soybeans, etc.) and the assessment of redundancy among existing ones—will be used to adaptively update the indicator set in Part I. This iterative loop ensures that subsequent versions of the anomaly detection model become increasingly focused on the environmental drivers most directly linked to AMR outcomes.

A further methodological consideration is that the SHAP interpretation, while highly informative, assumes feature independence during estimation and may not fully capture complex multivariate interactions. We anticipate that the planned Bayesian Network Analysis in Part II will directly overcome this constraint, as it is explicitly designed to model conditional dependencies and complex interactions between variables.

This planned study will integrate the key environmental drivers identified here with actual AMR measurements (e.g., MIC data) to:

1. Quantify Probabilistic Relationships: Model the conditional probabilities and the strength of influence each environmental driver has on AMR outcomes.
2. Incorporate Uncertainty: Explicitly account for uncertainty inherent in complex environmental and biological systems.
3. Develop Predictive Capability: Create a probabilistic model for predicting AMR risk based on environmental inputs, moving toward a true early-warning system.

This logical progression from unsupervised anomaly detection (Part I) to causal inference (Part II) is a powerful strategy for translating environmental signals into actionable insights. It provides a robust framework for fulfilling the promise of a One Health approach to combat AMR, where initial hypotheses generated from broad data are systematically validated and refined into a predictive, policy-ready tool.

## 5. Conclusions

This study underscores the potential of anomaly detection in environmental health surveillance. By flagging deviations from expected environmental patterns, our approach can prioritize high-risk areas for targeted AMR monitoring. The integration of SHAP values into anomaly detection enables not only the identification of outliers but also an understanding of the environmental context in which they occur. This multiscale interpretability is essential for informing risk-based monitoring strategies.

This study demonstrates that unsupervised anomaly detection—when paired with interpretable machine learning methods like SHAP—can uncover environmental patterns associated with AMR risk. Pesticide use, fertilizer intensity, land use change, and population density emerged as robust anomaly drivers, while other variables such as precipitation and specific crops showed localized but meaningful effects.

This scalable, data-driven strategy lays the groundwork for future early-warning systems that combine environmental and direct antimicrobial resistance data. Such systems could help bridge the gap between environmental signals and clinical or public health interventions, a connection we further plan to explore through complementary Bayesian analyses.

To this end, this research lays the essential foundation for a two-stage early-warning system. The unsupervised anomaly detection presented in this first stage successfully identifies aberrant environmental conditions without prior knowledge of AMR outcomes. These results form the basis for the next phase of this pipeline, which will employ a Bayesian network to rigorously test and quantify the probabilistic relationships between the identified environmental drivers and actual AMR prevalence, shifting the analysis from unsupervised pattern recognition to causal inference.

## Figures and Tables

**Table 1:** Selected indicators, data sources and their characteristics. The temporal coverage listed indicates the full range available from the source. For the analysis in this study, a standard period of 1990–2022 was used for all indicators where possible.

Indicator	Data Link	Variables	Temporal Coverage	Geographical Coverage	Use
Rapeseed Yield	https://www.fao.org/faostat/en/#data/QCL, accessed on 1 March 2025	Production volume, harvested area, yield (tonnes/ha)	1961–2023 (annual)	Global (country-level)	Biofuel feedstock and edible oil trends analysis
Maize Yield	https://www.fao.org/faostat/en/#data/QCL accessed on 1 March 2025	Production volume, harvested area, yield (tonnes/ha)	1961–2023 (annual)	Global (country-level)	Agricultural productivity and food security trends
Mean Surface Temperature Change	https://www.fao.org/faostat/en/#data/ET, accessed on 1 March 2025	Temperature change relative to 1951–1980 baseline	1961–2023 (annual, monthly, seasonal)	Global (country-level)	Long-term climatic trend assessment
Fertilizer Consumption (N, P, K)	https://www.fao.org/faostat/en/#data/RFN accessed on 1 March 2025	Fertilizer use by nutrient type (tonnes)	1961–2023 (annual)	Global (country-level)	Agricultural intensification and environmental impact analysis
Pesticide Use	https://www.fao.org/faostat/en/#data/RP, accessed on 1 March 2025	Insecticides, herbicides, fungicides	1990–2023 (annual)	Global (country-level)	Agrochemical use monitoring and environmental risk assessment
Precipitation	[ten00001] Water resources: long-term annual average	Annual precipitation (mm, million m^3^)	~1990–present (varies by country)	EU, EFTA, candidate countries	Water availability and climate variability assessment
Renewable Freshwater Resources	[ten00003] Fresh water abstraction by source per capita-m^3^ per capita	Internal/external flows, total renewable freshwater, per capita	~1990–present (varies by country)	EU, EFTA, candidate countries	Water sustainability and resource pressure analysis
Agricultural Land Use	https://ourworldindata.org/land-use, accessed on 1 March 2025	Arable land, permanent crops, pastures (hectares)	10,000 BCE–2023 CE (annual)	Global	Long-term land use change and agricultural expansion analysis
Population	https://www.fao.org/faostat/en/#data/OA, accessed on 1 March 2025	Sex, urban/rural status, projections	1950–2100 (annual)	Global (UN M49 classification)	Demographic analysis for food demand and environmental impact
Greenhouse Gas Emissions	[sdg_13_10] Domestic net greenhouse gas emissions	CO_2_, CH_4_, N_2_O, PFCs, HFCs, SF_6_, NF_3_	1990–latest (some data back to 1985)	EU Member States	Emission trends, mitigation, and policy evaluation

## Data Availability

The original contributions presented in this study are included in the article. Further inquiries can be directed to the corresponding author.

## Abbreviations

The following abbreviations are used in this manuscript:

AMR	Antimicrobial resistance
ARGs	Antimicrobial resistance genes
GHG	Greenhouse gas
iForest	Isolation Forest
KDE	Kernel Density Estimate
MDR	Multidrug-resistant
MICE	Multivariate Imputation by Chained Equations
MIC	Minimum inhibitory concentration
SHAP	Shapley Additive Explanations
t-SNE	t-distributed Stochastic Neighbor Embedding
WHO	World Health Organization
